# 

**Published:** 1881-12

**Authors:** 


### CONTENTS:

ART. I.—Proceedings of the Annual Meeting of the Dental Association of Maryland and District of Columbia, September 7th and 8th, 1881 .....337
ART. II.—The Newest of Our Creeds. By Prof. Hodgkin.....348
ART. III.—Principal Causes of Constitutional Derangement in First Dentition. By J. Hardman, D. D. S .....360
ART. IV.—Treatment of the Dental Pulp. By A. G. Bouton, D. D. S.....366
ART. V.—Dentists for the Army and Navy.....373

### EDITORIAL, ETC.

Patents and the Medical Men.....376

A Good Gold Foil.....377

### OBITUARY.

Dr. Daniel Harwood.....377

Dr. Joshua Tucker.....381

Dr. Joseph S. Hartman.....382

### BIBLIOGRAPHICAL.

Coles' Deformities of the Mouth.....383
The American Agriculturist.....383
Benedikt on Brains of Criminals.....383
The Scientific American.....384
Physicians' Visiting List for 1882.....384
The Sanitarian.....384
Sea Sickness.....384

